# Transcriptome Analysis Reveals Differentially Expressed Genes That Regulate Biosynthesis of the Active Compounds with Methyl Jasmonate in Rosemary Suspension Cells

**DOI:** 10.3390/genes13010067

**Published:** 2021-12-27

**Authors:** Deheng Yao, Zihao Zhang, Yukun Chen, Yuling Lin, Xuhan Xu, Zhongxiong Lai

**Affiliations:** Institute of Horticultural Biotechnology, Fujian Agriculture and Forestry University, Fuzhou 350002, China; yaodh337@126.com (D.Y.); zhangzihao863@163.com (Z.Z.); cyk68@163.com (Y.C.); buliang84@163.com (Y.L.); xxuhan@163.com (X.X.)

**Keywords:** *Rosmarinus officinalis* Lour., suspension cells, MeJA, antioxidant enzymes, RNA-seq, qRT-PCR, transcription factors

## Abstract

To study the effects of Methyl jasmonates (MeJA) on rosemary suspension cells, the antioxidant enzymes’ change of activities under different concentrations of MeJA, including 0 (CK), 10 (M10), 50 (M50) and 100 μM MeJA (M100). The results demonstrated that MeJA treatments increased the activities of phenylalanine ammonla-lyase (PAL), superoxide dismutase (SOD), peroxidase (POD), catalase (CAT) and polyphenol oxidase (PPO) and reduced the contents of hydrogen peroxide (H_2_O_2_) and malondialdehyde (MDA), thus accelerating the ROS scavenging. Comparative transcriptome analysis of different concentrations of MeJA showed that a total of 7836, 6797 and 8310 genes were differentially expressed in the comparisons of CKvsM10, CKvsM50, CKvsM100, respectively. The analysis of differentially expressed genes (DEGs) showed phenylpropanoid biosynthesis, vitamin B6, ascorbate and aldarate metabolism-related genes were significantly enriched. The transcripts of flavonoid and terpenoid metabolism pathways and plant hormone signal transduction, especially the jasmonic acid (JA) signal-related genes, were differentially expressed in CKvsM50 and CKvsM100 comparisons. In addition, the transcription factors (TFs), e.g., *MYC2*, *DELLA*, *MYB111* played a key role in rosemary suspension cells under MeJA treatments. qRT-PCR of eleven DEGs showed a high correlation between the RNA-seq and the qRT-PCR result. Taken together, MeJA alleviated peroxidative damage of the rosemary suspension cells in a wide concentration range via concentration-dependent differential expression patterns. This study provided a transcriptome sequence resource responding to MeJA and a valuable resource for the genetic and genomic studies of the active compounds engineering in rosemary.

## 1. Introduction

Rosemary (*Rosmarinus officinalis* Lour.) is a famous ornamental and medicinal homologous plant. Rosemary, as an excellent natural antioxidant and preservative, has been used in various industries widely [1]. Studies have shown that the main components of the active functions of rosemary include terpenoids, phenols, for example a new flavonoid 6′-O-(E)-feruloylhomoplantaginin, and the phenolic diterpene antioxidants (PDAs), carnosic acid and carnosol and rosmarinic acid [2,3,4]. The active ingredients of rosemary are widely used in anti-tumor, anti-cancer, anti-despondency, anti-virus, anti-inflammatory activity, regulating the immune system and other activities. Carnosic acid could alleviate H_2_O_2_ induced hepatocyte damage through the SIRT1 pathway and rosemary extract, and significantly up regulate the expression of *Nrf2* in colon cells and inhibit an HCT116 xenograft tumor formation in mice. Moreover, rosemary extract shows a higher antioxidant potential and increases the oxidative stability of oil by more than 30% compared to conventional synthetic antioxidants [5,6,7,8,9,10,11]. Researchers had previously attempted to regulate the synthesis of functional metabolites using various methods. MeJA as a signal molecule showed extensive regulations to the secondary metabolism, of which regulation was considered particularly important in changing the synthesis of plant functional metabolites in cells [12,13]. In addition, plant tissue and cell culture techniques were the most efficient methods for obtaining functional metabolites. Our group has established a rosemary suspension cells culture system to study the influence of MeJA treatment on functional metabolites.

MeJA had been used to elicit defense responses in many species through enhancing the secondary metabolites production [14,15], such as volatile terpenoids in *Amomum villosum*, triterpene in *Euphorbia pekinensis* and tropane alkaloids in *Hyoscyamus niger* [16,17,18]. An analysis of transcriptome after MeJA treatment could find key genes involved in the biosynthesis of active compounds and be used to unveil the relation between genes and metabolism [19,20]. In *Catharanthus roseus*, the MeJA-responsive expression of terpenoid indole alkaloids biosynthesis genes was controlled by protein *CrMYC2*’s regulation of *ORCA* gene expression, regulating a series of terpenoid indole alkaloids biosynthesis genes [21]. The TFs AP2/ERF and bHLH cooperatively mediate jasmonate-elicited nicotine biosynthesis, which via the JA induced signaling cascade leads to increased nicotine biosynthesis in *Tobacco* [22,23]. In *Artemisia annua* suspension cells, exogenous MeJA induced the expression of *CYP71AV1* and promoted the accumulation of artemisinin [24].

MeJA treatment can induce the biosynthesis of many secondary metabolites (terpenoids, phenylpropanoids) and acts as an elicitor of secondary metabolite production across the plant kingdom [25]. *SlMYC1* acts synergistically with *SlEOT1* in the transactivation of the *SlTPS5* promoter to induce the biosynthesis of terpene in *Solanum lycopersicum* with JA treatment [26]. There were 13 predicted genes that could participate in the biosynthesis of flavonoids under MeJA treatment in *E. breviscapus* [27]. Transcript analysis suggested that MeJA up-regulated transcripts of terpenoids and flavonoids, *ObAS1* and *ObAS2* were identified and characterized in *Sweet Basil* [28]. MeJA treatment revealed differential expression of genes involved in phenylpropanoid biosynthesis (*IiPAL*, *IiC4H, Ii4CL*), and lignin biosynthesis (*IiCAD*, *IiC3H*, *IiCCR*, *IiDIR* and *IiPLR*), and 112 putative AP2/ERF TFs in *Isatis indigotica* [29]. Based on the gene annotation of the transcriptome, 104 unigenes were identified and their responses to MeJA induction were investigated involved in the biosynthesis of indole, terpenoid and phenylpropanoid [30]. The results showed that transcriptional levels of *SgHMGR*, *SgSQS*, *SgCS* and *SgCYP450* were up-regulated and their responses in the presence of MeJA were related to the concentration and timing of MeJA treatment in *Siraitia grosvenorii* [31]. MeJA-regulated rubber biosynthesis, based on a differential expression analysis, showed that the expression of *HMGCR*, *FPPS*, *IDI*, *GGPPS*, *REF/SRPP* and transcription factors (bHLH, MYB, AP2/EREBP and WRKY) increased with MeJA treatment in TKS [32]. Based on the date of the transcriptome in *Taxus*, there were 18 genes showing increased transcript abundance following elicitation of MeJA, which was involved in the biosynthesis of terpenoid backbone, and then multiple candidates for the unknown steps in paclitaxel biosynthesis were identified [33]. A total of 40,952 unigenes and 19 coumarin compounds, 7 cytochrome-P450, 8 multidrug resistance transporter unigenes and 8 marker compounds were obtained, involved in coumarins biosynthesis and transport pathway with a parallel analysis of transcript and metabolic profiles in *Peucedanum praeruptorum* [34]. The secondary metabolites are the main components of their active functions in plant. Therefore, transcriptome technology was widely used as an effective means to research the biosynthesis of active compounds and the mining of key enzyme genes. MeJA could promote the biosynthesis of terpenoids and phenylpropanoids, through revealing differential expression of the genes involved in biosynthesis and plant hormone signal transduction and TFs in plants.

At present, no study has shown the transcriptome of rosemary responding to MeJA. In our study, we investigated the transcriptome of rosemary suspension cells responding to different concentrations of MeJA using high-throughput sequencing technologies. Putative gene expression profiles of rosemary suspension cells were investigated and DEGs were classified under different concentrations of MeJA. By comparing and analyzing the sequencing data of control and illuminated groups, the genes involved in the regulation of primary and secondary metabolism and their regulatory networks were established. These experiments reveal dynamic gene expression changes in responding to different concentrations of MeJA and provide new insights into the genetic and genomic regulation of plant functional metabolites.

## 2. Materials and Methods

### 2.1. Plant Material and MeJA Treatments

Rosemary callus was obtained by the following methods: treating rosemary leaves with 0.1% mercury bichloride solution, then cutting the leaves into small pieces about 0.5 cm × 0.5 cm, using the inoculation method that the back of leaves contacts with the medium, on Murashige and Skoog (MS) medium (30 g/L sucrose, pH 5.8) with 0.5 mg/L 1-Naphthaleneacetic acid (NAA) and 4.0 mg/L N-(Phenylmethyl)-9H-purin-6-amine (6-BA). After the suspension culture for several generations in the MS liquid medium supplemented with 1.0 mg/L 2,4-dichlorophenoxyacetic acid (2,4-D), which lasted for 8 days at 25 ± 0.5 °C with shaker speed 120 rpm in the dark, it could derive rosemary suspension cell lines with high cell viability and stable growth. The culture was performed by transferring 4 g·FW/20 mL of 6-day-old culture (cells plus medium) to 80 mL of the fresh growth medium, which lasted for 8 days. MeJA were sterilized and added to the medium on day 6 of the culture process. Baes on the pre-experiment, 10, 50 and 100 μM MeJA treatments could promote the accumulation of rosmarinic acid, carnosic acid and flavonoids in rosemary suspension cells, so we chose the concentration of MeJA solution was 0, 10, 50 and 100 μM. Each test was repeated three times. After 48 h treatment, all materials were stored at −80 °C for later use.

### 2.2. RNA-Seq Library Construction

Total RNA was isolated from rosemary suspension cells (three replicates) with the RNAprep Pure Plant Kit (TIANGEN, Beijing, China). The integrity and concentration of the RNA samples were further measured using an Agilent 2100 Bioanalyzer (Agilent, CA, USA) and the purity of the RNA samples was assessed using the NanoPhotometer® spectrophotometer (NP80, IMPLEN, Munich, Germany). RNA libraries were prepared using the True-seq RNA sample preparation kit according to the manufacturer’s instructions. The constructed library was tested on the Agilent 2100 Bioanalyzer and the ABI StepOnePlus Real-Time PCR System. Finally, mRNA libraries were sequenced on an Illumina HiSeq 4000 platform (Shenzhen, China).

### 2.3. Sequencing, Assembly and Annotation of the Transcriptome

The cDNA libraries of four samples were sequenced by 2 × 100 bp paired-end sequencing on an Illumine HiSeq platform according to the manufacture’s instructions. We used Trinity to assemble clean reads by de novo, while removing PCR duplication to improve assembly efficiency. Then the assembled transcripts were clustered by tgicl to remove redundancy and obtain UniGene. Trinity consists of three independent modules: inchworm, chrysalis and butterfly, which process a large number of reads in turn. The assembled UniGene will be annotated with seven functional databases (KEGG (Kyoto Encyclopedia of Genes and Genomes), GO (Gene Ontology), NR, NT, Swissprot, Pfam and KOG).

### 2.4. Quantification of Gene Expression Levels

We used bowtie2 to align clean reads to the genome sequence and RESM (http://deweylab.biostat.wisc.edu/rsem/rsem-calculate-expression.html, Access date: 30 January 2019, RESM Version: v1.2.8; RESM Parameter: default;) to calculate the gene expression level of each sample. RESM is a software package for RNA-seq reads to calculate the expression of genes and transcript subtypes.

### 2.5. Differential Expression Genes Analysis

Degseq method is based on Poisson distribution. In this project, DEG detection is carried out according to the method described by Wang et al. 2010 [35]. Differential expression analysis of the four treatments was performed using the DEGSeq R package. *p*-Values were adjusted using the Benjamini and Hochberg method. A corrected P-value (false discovery rate, FDR) of 0.001 and a fold change of 2 were set as the default threshold for defining significant differential expression.

### 2.6. GO and KEGG Enrichment Analyses of Differentially Expressed Genes

According to the GO (http://www.geneontology.org/, Access date: 30 January 2019) and KEGG (http://www.genome.jp/kegg/, Access date: 30 January 2019) annotation results and the official classification, DEGs were classified, and the enrichment factors were analyzed using the phyper function in R software (https://en.wikipedia.org/wiki/Hypergeometric_distribution, Access date: 30 January 2019).

### 2.7. Validation of the DEGs by qRT-PCR

To validate the RNA-Seq results, 11 DEGs were subjected to qRT-PCR analysis performed on the LightCycler480 real-time PCR system (Roche, Basel, Switzerland) in a 20 µL final volume containing 10 µL of 2 × SYBR Premix Ex TaqTM (Takara, Shanghai, China), 1 µL of 10 × diluted cDNA, and 0.8 µL specific primer pairs, and 7.4 µL of ddH_2_O. The changes in mRNA expression were calculated using the comparative 2^−∆∆Ct^ method. Specific primers were designed using DNAMAN V6.0; the primer pair sequences are listed in Table S1. All treatments were analyzed in three biological replicates.

### 2.8. Measurement of Antioxidant Enzymes and Non-Enzymatic Antioxidants

Briefly, 0.1 g of rosemary suspension cells (fresh weight) were taken from each of the treatment groups and rapidly frozen with liquid nitrogen. The samples were maintained at 2–8 °C after melting. Then, 1 mL PBS (pH 7.4) was added, followed by homogenization by hand or grinders and centrifugation for 20 min at the speed of 2000–3000 rpm. Supernatant was removed. The activities of PAL, SOD, POD, CAT, PPO and the contents of MDA, H_2_O_2_, proline (pro) were assayed using ELISA Kit (Weilan, Shanghai, China) and a micro-plate reader (Rayto RT-6100) according to the manufacturer’s instructions. The principle of the assay: the kit assay plant PAL, SOD, POD, CAT, PPO, MDA, H_2_O_2_ and proline level in the sample, use purified plant PAL, SOD, POD, CAT, PPO, MDA, H_2_O_2_ and proline antibody to coat microtiter plate wells, make solid-phase antibody, then add PAL, SOD, POD, CAT, PPO, MDA, H_2_O_2_ and proline to the wells. Combined antibody labeled with HRP became an antibody-antigen-enzyme-antibody complex. After washing completely, TMB substrate solution was added. TMB substrate became blue. At HRP catalyzed enzyme, the reaction is terminated by the addition of a sulphuric acid solution and the color change is measured spectrophotometrically at a wavelength of 450 nm. The concentration of PAL, SOD, POD, CAT, PPO, MDA, H_2_O_2_ and proline in the samples is then determined by comparing the OD of the samples to the standard curve. Assay procedure: add standard, set standard wells, test sample wells, add standard 50 μL to standard well; add sample, set blank wells separately, test sample well, add sample dilution 40 μL to test sample well, then add testing sample 10 μL (sample final dilution is 5-fold), add sample to wells, do not touch the well wall as far as possible, and gently mix; add enzyme, add HRP-conjugate reagent 100 μL to each well, except blank well; incubate, after closing the plate with closure plate membrane, incubate for 60 min at 37 °C; configurate liquid, 20-fold wash solution diluted 20-fold with distilled water and reserve; wash, uncover the closure plate membrane, discard liquid, dry by swinging, add washing buffer to every well, still for 30 s then drain, repeat 5 times, dry by patting; color, add chromogen solution A 50 uL and B to each well, evade the light preservation for 15 min at 37 °C; stop the reaction, add stop solution 50 μL to each well, stop the reaction (blue color changes to yellow); assay, take blank well as zero, read absorbance at 450 nm after adding stopping solution within 15 min.

### 2.9. Statistical Analysis

Quantitative results for rosemary metabolite content, enzyme activity and gene expression analyses are presented as the means ± standard deviations (SDs) of at least three biological replicates. The effects of MeJA conditions on metabolite contents and gene expression were analyzed by one-way analysis of variance (ANOVA) followed by Duncan’s test using SPSS version 19.0. Figures were prepared using GraphPad Prism 8.0 and Excel 2016 software.

## 3. Results

### 3.1. Physiological and Biochemical Indexes of Rosemary Suspension Cells under Different Concentrations of MeJA

**Figure 1:**
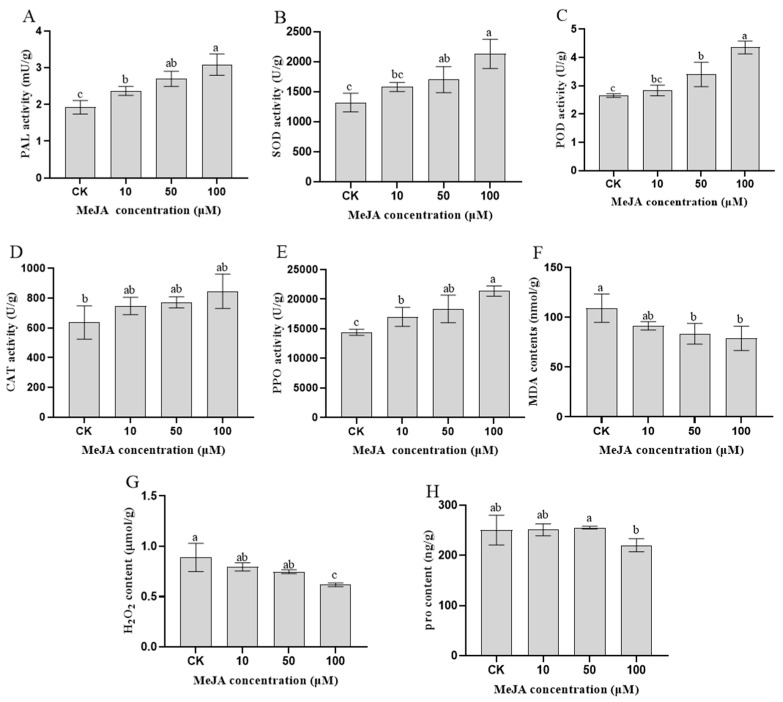
Physiological and biochemical indicators of rosemary suspension cells under different concentrations of MeJA. (**A**) changes in PAL activities. (**B**) changes in SOD activities. (**C**) changes in POD activities. (**D**) changes in CAT activities. (**E**) changes in PPO activities. (**F**) changes in MDA contents. (**G**) changes in H_2_O_2_ contents. (**H**) changes in proline contents. Values represent means ± SDs of three replicates. Different lower-case letters indicate statistically significant differences at the 0.05 level by one-way ANOVA with Duncan’s test.

In our study, measuring the physiological and biochemical indicators in rosemary suspension cells is helpful for understanding how the synthesis of important metabolites is promoted by MeJA. After different concentrations of MeJA treatment for 48 h in rosemary suspension cells, the activities of PAL were the highest in the 100 μM MeJA treatment group, followed by the 50 and 10 μM MeJA treatment groups, they were higher than the CK treatment (Figure 1A). Similarly, the activities of SOD, POD, CAT and PPO were the highest in the 100 μM MeJA treatment group, followed by the 50 and 10 μM MeJA treatment groups, they were higher than the CK treatment (Figure 1B–E). The concentration of MDA and H_2_O_2_ were the lowest in the 100 μM MeJA treatment group, the highest of the concentration was the CK treatment (Figure 1F,G). For the proline contents, the highest was the 50 μM MeJA treatment group and the lowest was the 100 μM MeJA treatment group (Figure 1H). The result indicating that the MeJA activated the rosemary suspension cells enzyme antioxidant system accelerate the ROS scavenging, the antioxidant enzyme activity increased with the increase of MeJA concentration and had a key role in rosemary suspension cells. 

### 3.2. RNA-Seq Analysis of Rosemary Suspension Cells

To study the effects of different concentrations of MeJA on rosemary suspension cells at a transcriptional level, four mRNA libraries were constructed and sequenced from the four concentrations of MeJA treatment (CK_(1,2,3), M10_(1,2,3), M50_(1,2,3) and M100_(1,2,3)). After removing the linker sequence, the RNA-Seq data of the twelve rose-mary suspension cells libraries produced 66,314,608 to 71,191,500 reads, respectively, due to differences in concentrations of MeJA. The values of Q20 were higher than 97% in all the samples, indicating the high reliability of the rosemary suspension cells transcriptome sequencing data (Tables S2–S4).

In addition, unigenes were exposed to Nr, Nt, SwissPort, Pfam, KEGG, GO and KOG databases using BLAST analysis (E-value < 0.00001) (Table S5). The unigenes annotated in the Nr database were counted, the top five species were annotated: *Sesamum indicum* L. (65.02%), *Erythranthe guttata* L. (17.59%), *Dorcoceras hygrometricum* L. (2.08%), *Salvia miltiorrhiza* L. (2.08%) and *Ipomoea nil* L. (0.75%). The 17.59% and 2.08% of the total number of Nr annotations accounted for, respectively, 2.08% and 0.75% (Figure S1). The distribution of gene functions in GO was grouped into biological processes, cellular component and molecular function, cellular process (14,755 unigenes), cell (14,796 unigenes), binding (25,721 unigenes) and catalytic activity (23,776 unigenes) were dominant subcategories (Figure S2). Similarly, KOG functional classification showed that the uppermost classification was general function prediction only (15,177 unigenes) followed by signal transduction mechanisms (9078 genes). In addition, 1205 unigenes were annotated for secondary metabolites biosynthesis, transport and catabolism (Figure S3). At last, unigenes were also annotated against KEGG database for understanding advanced-level utilities and functions of the biological structure. Among 80,961 unigenes were annotated in 136 pathways, metabolism (102 pathways, 40,920 unigenes) was the most significant category, a substantial number of genes were related to carbohydrate metabolism (6277 genes) and amino acid metabolism (3835 gens) (Figure S4).

### 3.3. Global Analysis of Gene Expression across the Four Distinct Samples under Different Concentrations of MeJA

**Figure 2:**
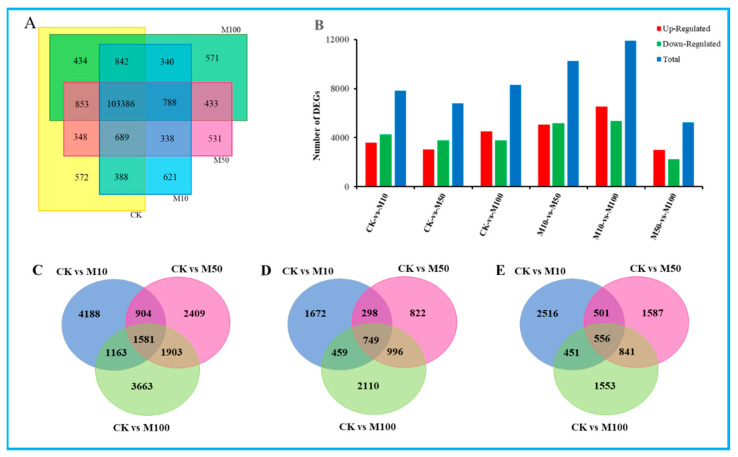
Statistical analysis of differentially expressed unigenes in rosemary suspension cells under different concentrations of MeJA. (**A**) The Venn diagram of expressed genes in four MeJA treatments. (**B**) statistic of up/down-regulated genes in pairwise comparisons. (**C**) Venn diagram of DEGs under MeJA treatment. (**D**) Venn diagram of the unique and common regulated DEGs up-regulated of DEGs under MeJA treatments. (**E**) Venn diagram of the unique and common regulated DEGs down-regulated of DEGs under MeJA treatments.

In our study, there were 107,512, 102,762, 107,366 and 107,646 expressed genes in CK, M10, M50 and M100 sample libraries. Among these, 103,386 expressed genes were present in all four sample libraries. However, only 572, 621, 531 and 571 genes were uniquely present in CK, M10, M50 and M100 sample libraries, respectively (Figure 2A), which suggested that distinct spatial transcriptional patterns were present in the sample libraries. In order to evaluate the differences in molecular response among four samples, gene expression was normalized to FPKM. After filtering with FPKM> 10, a total of 14,104 (13.11%), 13,880 (12.87%), 13,981 (12.97%) and 13,940 (12.95%) genes were expressed in CK, M10, M50 and M100, respectively (Table S6). The top 20 most enriched (FPKM) genes ranged from 682 to 3814, 781 to 3294, 663 to 3844 and 709 to 4294, respectively. The top 20 most expressed genes from the four libraries were shown as *defensin-like cystein-rich peptide*, *extracellular ribonuclease LE, PREDICTED: titin-like*, *pathogen-related protein STH-2, hypothetical protein SELMODRAFT_431225, aquaporin-like protein, partial, major pollen allergen Lol p 11-like and extensin-3*—highly expressed in CK, M10, M50 or M100 sample libraries (Table S7). 

To further understand the changes in the rosemary suspension cells transcriptome under different concentrations of MeJA, Poisson D was used to calculate the expression level of each gene. We filtered the DEGs with |log2fold change| ≥ 1 and FDR < 0.001 between these six pairs the comparisons were as follows: CKvsM10, CKvsM50, CKvsM100, M10vsM50, M10vsM100, M50vsM100, among these contained 7836, 6797, 8310, 10,240, 11,890 and 5260 DEGs, respectively, which included 3596, 325, 4528, 5070, 6537 and 3017 up-regulated genes and 4240, 3772, 3782, 5170, 5353 and 2243 down-regulated genes (Figure 2B). The comparative analysis of the CKvsM10, CKvsM50, and CKvsM100 by Venn diagram showed that 1581 genes were commonly differentially expressed, and 4188, 2409 and 3663 genes were unique to each comparison, respectively; there were 749 genes commonly up-regulated, while 1672, 822 and 2110 genes in each comparison were uniquely up-regulated; similarly, there were 556 genes commonly down-regulated, while 2516, 1587 and 1553 genes in each comparison were uniquely down-regulated, respectively (Figure 2C–E). These DEGs’ analysis results revealed that rosemary suspension cells transcriptome undergoes significantly dynamic changes under different concentrations of MeJA. Therefore, the transcriptome datasets of rosemary suspension cells under different concentrations of MeJA might serve as a valuable molecular resource for future studies.

### 3.4. GO Enrichment Analysis of DEGs in Rosemary Suspension Cells

**Figure 3:**
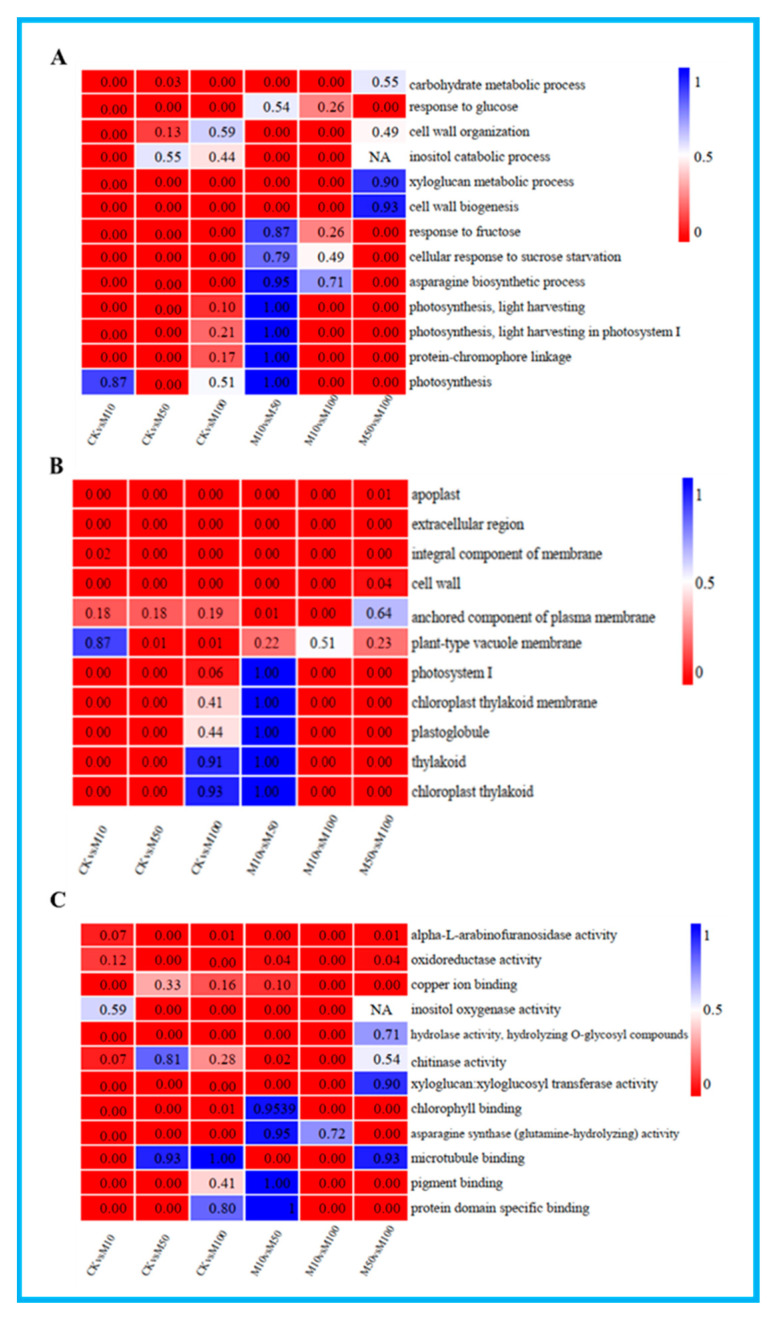
GO-terms enrichment analysis. From the red to blue corresponds to the numerical value of corrected Q and significant enriched GO-terms from the low to the high. (**A**) biological process; (**B**) cellular component; (**C**) molecular function.

To further understand the potential functions of the DEGs under different concentrations of MeJA, GO terms assignment to classify the functions of DEGs was performed in pairwise comparisons under three GO main categories: biological process, cellular component and molecular function (Table S8). The results of GO enrichment analysis in all comparisons showed the enrichment of most biological processes in the CKvsM10 and CKvsM50 combinations were significantly higher than other combinations, DEGs were mainly related to xyloglucan metabolic process and response to glucose in the CKvsM10, CKvsM50 and CKvsM100 comparisons (Figure 3A). The enrichment of most cellular components in the CKvsM50 combination was significantly higher than other combinations, DEGs were mainly related to apoplast and cell wall in the CKvsM10, CKvsM50 and CKvsM100 comparisons (Figure 3B). The enrichment of most molecular functions in the CKvsM10 combination was significantly higher than other combinations, DEGs were mainly related to hydrolase activity, alpha-L-arabinofuranosidase activity and oxidoreductase activity in the CKvsM10, CKvsM50 and CKvsM100 comparisons (Figure 3C). In summary, indicating that the effect of different concentrations of MeJA on rosemary suspension cells was particularly obvious and had substantially different responses to different concentrations of MeJA.

### 3.5. KEGG Enrichment and Mapman Analysis of DEGs in Rosemary Suspension Cells

**Figure 4:**
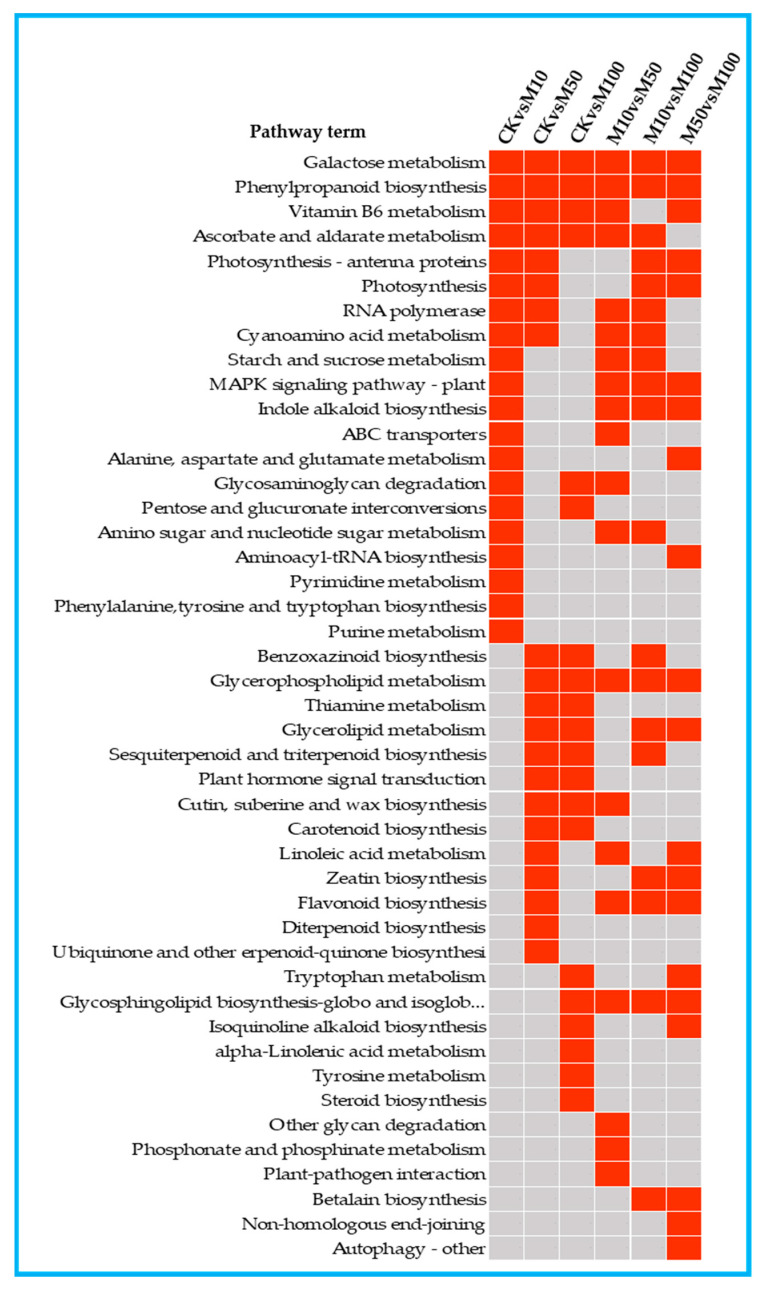
The top 20 KEGG pathways enriched by DEGs in in the comparisons. Red indicates significant enrichment and gray indicates no significant enrichment.

To investigate the metabolic pathways of the DEGs, we used the KEGG database to classify the DEGs function and mapped the top 20 KEGG pathways enriched among the DEGs according to the enrichment factors identified (Figures S4 and S5). The first 20 enrichment pathways of the six combinations (CKvsM10, CKvsM50, CKvsM100, M10vsM50, M10vsM100, M50vsM100) were compared and analyzed (Figure 4). Phenylpropanoid biosynthesis was enriched in the top 5 in the CKvsM10, CKvsM50, CKvsM100, M10vsM50 and M10vsM100 comparisons. Phenylpropanoid biosynthesis is closely related to the biosynthesis of flavonoids, and phenylalanine is closely related to the synthesis of rosmarinic acid, indicating that there were significant differences in the biosynthesis of flavonoids and rosmarinic acid in rosemary suspension cells by different concentrations of MeJA.

The first 20 enrichment pathways of the three combinations (CKvsM10, CKvsM50, CKvsM100) were compared and analyzed (Figure 4). Galactose metabolism, phenylpropanoid biosynthesis, vitamin B6 metabolism, ascorbate and aldarate metabolism were enriched in the three comparisons among the top 20 pathways (Figure 4). This indicated that these pathways differed significantly under different MeJA concentrations treatments. Some pathways were enriched in the top 20 in two combinations, photosynthesis-antenna proteins, photosynthesis and RNA polymerase were enriched in the top 20 in CKvsM10 and CKvsM50 (Figure 4). This indicated that these pathways might play important roles in responding to 10 and 50 μM MeJA treatments. Glycosaminoglycan degradation and pentose and glucuronate interconversions were enriched in the top 20 in CKvsM10 and CKvsM100 (Figure 4), suggesting that 10 and 100 μM MeJA were more effective for them. Benzoxazinoid biosynthesis, sesquiterpenoid and triterpenoid biosynthesis, plant hormone signal transduction and carotenoid biosynthesis were enriched in the top 20 in CKvsM50 and CKvsM100 (Figure 4), suggesting that 50 and 100 μM MeJA were more effective for these pathways. Some pathways were only enriched in the top 20 for one combination (Figure 4). MAPK signaling pathway-plant, indole alkaloid biosynthesis, ABC transporters, and phenylalanine, tyrosine and tryptophan biosynthesis were only enriched in the top 20 in CKvsM10. Linoleic acid metabolism, zeatin biosynthesis, flavonoid biosynthesis, diterpenoid biosynthesis and ubiquinone and other terpenoid-quinone biosynthesis were only enriched in the top 20 in CKvsM50. Tryptophan metabolism, isoquinoline alkaloid biosynthesis, alpha-Linolenic acid metabolism, tyrosine metabolism and steroid biosynthesis were only enriched in the top 20 in CKvsM100, indicating that 50 and 100 μM MeJA were more effective than 10 μM MeJA for metabolites such as terpenoids and flavonoids.

Mapman analysis of the DEGs was distributed in the cell wall, lipids, ascorbate and glutathione, sucrose and amino acid pathways (Figure S6A–C). More up-regulated DEGs were identified in the CKvsM100 comparison than the CKvsM10 and CKvsM50 comparisons in lipids, ascorbate and glutathione and amino acid pathways. More DEGs were identified in the CKvsM50 and CKvsM100 than the CKvsM100 comparison in the flavonoid metabolism pathway and more numbers of up-regulated DEGs were identified in the CKvsM50 and CKvsM100 comparisons. More DEGs were identified in the CKvsM10 and CKvsM100 than the CKvsM50 comparison in Non-MVA, MVA and phenylpropanoids pathways (Figure S6D–F). The above results indicate that 100 μM MeJA affected the metabolism including amino acids lipids, ascorbate, glutathione terpenoids and total flavonoids significantly more than 10 and 50 μM MeJA in rosemary suspension cells.

GO and KEGG enrichment and Mapman analyses of the DEGs showed that MeJA affected the synthesis of rosemary suspension cells’ metabolites via multiple pathways, including plant hormone signal transduction, reactive oxygen species (ROS) clearance, osmotic balance, phenylpropanoid biosynthesis and many other metabolism pathways that may play important regulatory roles.

### 3.6. Differential Expression Analysis of Plant Hormone Signal Transduction Related Genes during Rosemary Suspension Cells under Different Concentrations of MeJA

To reveal the potential key genetic factors in rosemary suspension cells under different concentrations of MeJA, we found a total of 16 DEGs were assigned to JA biosynthesis, including 1 *LOX2S*, 2 *AOS*, 1 *AOC*, 2 *OPR*, 1 *OPCL1*, 7 *ACX*, 1 *MEP2* and 1 *J-O-MT*. *LOX2S*, *OPCL1* and *ACX* (Unigene8348_All, Unigene10587_All) were up-regulated from 0 to 100 μM MeJA treatments, *OPR* (Unigene16468_All) and *ACX* (Unigene3995_All, Unigene5819_All) were down-regulated from 0 to 100 μM MeJA treatments. *AOS* was down-regulated under 10 μM MeJA treatment but up-regulated under 100 μM MeJA treatment. Other DEGs showed different expression patterns in the three combinations (Figure S7A).

Meanwhile, we found a total of 25 DEGs were assigned to JA signal transduction, including 2 *JAR1*, 1 *COI1*, 9 *JAZ* and 13 *MYC2*. Only 1 *JAR1* (Unigene18877_All) was up-regulated under 10 μM MeJA treatment, others were down-regulated. *COI1* was drastically up-regulated from 0 to 100 μM MeJA treatment. *JAZ* (CL9927.Contig1_All) was down-regulated under 10 μM MeJA treatment, others were up-regulated. 1 *MYC2* (CL4564.Contig5_All) was down-regulated from 0 to 100 μM MeJA treatment, 6 *MYC2* (CL2762.Contig2_All, CL4726.Contig3_All, CL7026.Contig2_All, CL9744.Contig1_All, CL12032.Contig4_All, Unigene30434_All) were down-regulated from 0 to 100 μM MeJA treatments, the number of up-regulated DEGs under 100 μM MeJA treatment were the most, secondly was 50 μM MeJA treatment, the least was 10 μM MeJA treatment (Figure S7B). The result showed that 100 μM MeJA treatment can significantly induce the DEGs expression of JA biosynthesis and signal transduction pathway compared to10 and 50 μM MeJA, indicating that 100 μM MeJA could significantly affect JA biosynthesis and signal transduction in rosemary suspension cells.

In our study, based on the KEGG and other annotation, plant hormone signal transduction was the representative pathway. There were 248 significant DEGs were assigned to the plant hormone signal transduction pathway involved in auxin (59 DEGs), cytokinine (56 DEGs), gibberellin (GA, 34 DEGs), abscisic acid (ABA, 15 DEGs), ethylene (ET, 44 DEGs), brassinosteroid (BR, 32 DEGs), and salicylic acid (SA, 8 DEGs) (Figure S7C–I). The result of the analysis of these DEGs showed that 69 DEGs were up-regulated under different concentrations of MeJA, including 4 *AUX1*, 3 *TIR1*, 14 *AUX-IAA*, 5 *ARF* and 8 *TF*, 87 DEGs were down-regulated, for example, 1 *SAUR*, 10 *TF*, 22 *B-ARR*, 4 *A-ARR*, 3 *ABF*, 6 *ETR* and 3 *CTR1*. The result also showed that 50 and 100 μM MeJA could significantly inhibit the DEGs expression of the salicylic acid signal transduction pathway (Figure S7C). There were more numbers of up-regulated DEGs in auxin and ABA signal transduction under different concentrations of MeJA (Figure S7D,E), but more numbers of down-regulated DEGs in other hormone signals transduction pathways under different concentrations of MeJA (Figure S7F–I).

### 3.7. Phenylpropanoid Biosynthesis Related Genes Were Differential Expressed during Rosemary Suspension Cells under Different Concentrations of MeJA

**Figure 5:**
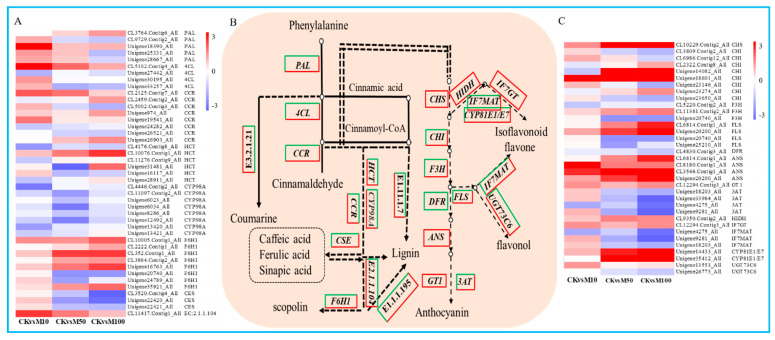
DEGs assigned to phenylpropanoid and flavonoid biosynthesis pathways under MeJA treatments. (**A**) heat map of the expression of DEGs in phenylpropanoid biosynthesis. (**B**) simplified diagram of phenylpropanoid and flavonoid biosynthetic pathway. (**C**) heat map of the expression of DEGs in flavonoid biosynthesis.

Phenylpropanoid biosynthesis was the representative KEGG pathway, we found 138, 104 and 138 DEGs assigned to phenylpropanoid biosynthesis in CKvsM10, CKvsM50 and CKvsM100 combinations, respectively (Figure 5B). These DEGs include 5 *PAL*, 4 *4CL*, 8 *CCR*, 6 *HCT*, 8 *C3’H* (*CYP98A*), 8 *F6H1*, 3 *CSE*, 1 *caffeoyl-CoA-O-methyltransferase*, 21 *β-glucosidase*, 27 *cinnamyl-alcohol dehydrogenase*, 66 *peroxidase*. There were more numbers of up-regulated DEGs than down-regulated DEGs under 10 μM MeJA treatments, but there were more numbers of down-regulated DEGs under 50 and μM MeJA treatment. Among these DEGs, *PAL* (CL3764.Contig6_All), *4CL* (CL5102.Contig4_All), *CCR* (Unigene974_All), *HCT* (CL10076.Contig1_All), *F6H1* (CL352.Contig1_All), peroxidase (Unigene16028_All), *β-glucosidase* (CL41.Contig21_All), *caffeoyl-CoA-O-methyltransferase* (CL11417.Contig1_All), *cinnamyl-alcohol dehydrogenase* (CL5952.Contig4_All) were drastically up-regulated from 0 to 100 μM MeJA treatment (Figure 5A and Figure S8). The result showed that 10 μM MeJA treatment can significantly induce the DEGs expression of phenylpropanoid biosynthesis compared to 50 and 100 μM MeJA in rosemary suspension cells. 

To reveal the potential key genetic factors in rosemary suspension cells under different concentrations of MeJA, we found a total of 35 DEGs were assigned to flavonoids biosynthesis (Figure 5B). Among these DEGs, these were 14 DEGs drastically up-regulated under different concentrations of MeJA treatments, include 1 *CHS*, 1 *CHI*, 1 *F3H*, 2 *FLS*, 4 *ANS*, 1 *GT1*, 1 *HIDH*, 1 *IF7GT*, 1 *CYP81E1/E7*, 1 *UGT73C6*. Instead, these were 5 DEGs drastically down-regulated from 0 to 100 μM MeJA treatment, include 1 *F3H*, 1 *DFR*, 1 *3AT*, 1 *IF7MAT* and 1 *UGT73C6*. The other 16 DEGs showed different differential expression patterns under different concentrations of MeJA, most of the DEGs were up-regulated under 10 μM MeJA treatment, but down-regulated under 50 and 100 μM MeJA treatment, for example, *CHI* (Unigene14082_All), which was down-regulated more significant under 100 than 50 μM MeJA treatment (Figure 5C). Therefore, indicating that different concentrations of MeJA could significantly affect the accumulation of flavonoids in rosemary suspension cells.

### 3.8. Terpenoid Biosynthesis Related Genes Were Differential Expressed during Rosemary Suspension Cells under Different Concentrations of MeJA

**Figure 6:**
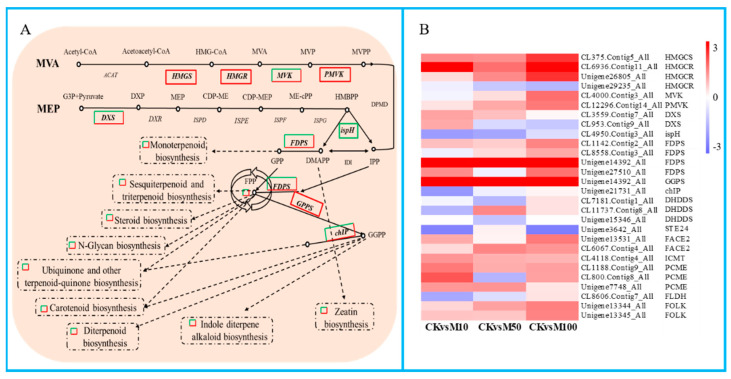
DEGs assigned to terpenoid biosynthesis pathway under MeJA treatments. (**A**) simplified diagram of the terpenoid biosynthesis pathway. (**B**) heat map of the expression of DEGs related to terpenoid backbone biosynthesis pathway.

In our study, the results showed that a large number of DEGs were found in the terpenoid biosynthesis and terpenoids biosynthesis pathways (Figure 6). There were 6 significant DEGs assigned to the MVA pathway, including 1 *HMGCS*, 3 *HMGCR*, 1 *MVK*, and 1 *PMVK*. Among these DEGs except *HMGCS* (CL375.Contig4_All) and *HMGCR* (Unigene29235_All, CL6936.Contig10_All) were drastically up-regulated under different concentrations of MeJA treatments. There were 3 significant DEGs that were assigned to the MEP pathway, including 2 *DXS* and 1 *ispH*. *DXS* were down-regulated under 10 μM MeJA treatment, but up-regulated under 50 and 100 μM MeJA treatments, ispH was drastically down-regulated under different concentrations of MeJA treatments. The result showed that 100 μM MeJA treatment could significantly induce the DEGs expression of MVA biosynthesis compared to 50 and 100 μM MeJA, 10 μM MeJA treatment could significantly inhibit the DEGs expression of MVA biosynthesis in rosemary suspension cells. In addition, the DEGs *FPS* (*FDPS*) and *GGPS,* which directly act on terpenoids to synthesize precursors geranyl diphosphate (GPP), farnesyl pyrophosphate (FPP) and geranylgeranyl pyrophosphate (GGPP) and other DEGs in the terpenoid biosynthesis pathway showed differential expression patterns under different concentrations of MeJA, for example *FDPS* (CL1142.Contig2_All, Unigene14392_All), *GGPS*, *FACE2*, *ICMT*, *PCME* (Unigene7748_All) and *FOLK* were up-regulated from 0 to 100 μM MeJA treatment. We also found a large number of DEGs were assigned to ubiquinone and other terpenoid-quinone biosynthesis, carotenoid biosynthesis, sesquiterpenoid and triterpenoid biosynthesis, zeatin biosynthesis, steroid biosynthesis, monoterpenoid biosynthesis, diterpenoid biosynthesis and N-Glycan biosynthesis pathways (Figure S9). Therefore, different concentrations of MeJA could significantly affect the accumulation of terpenoids in rosemary suspension cells.

### 3.9. Transcription Factors Are Important in Rosemary Suspension Cells under Different Concentrations of MeJA

**Figure 7:**
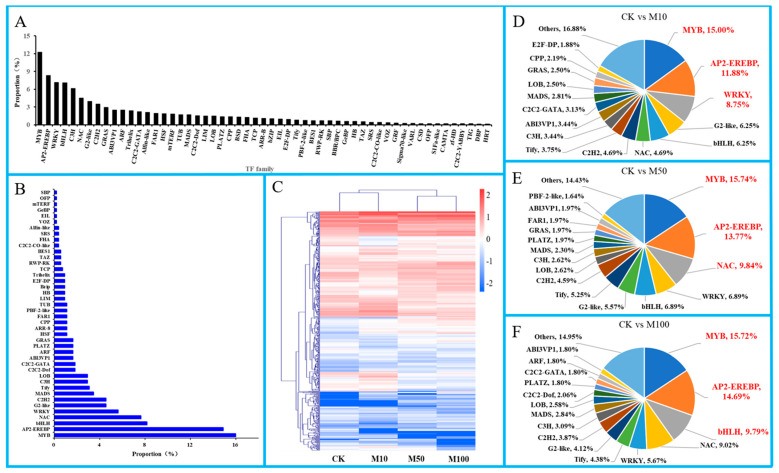
TFs in rosemary suspension cells under MeJA treatments. (**A**) classification statistics of TFs. (**B**) the Frequency distribution of differentially expressed TFs. (**C**) cluster analysis of expression profiles of TFs. (**D**) statistic of differentially TFs in the comparisons of CKvsM10. (**E**) statistic of differentially expressed TFs in the comparisons of CKvsM50. (**F**) statistic of differentially expressed TFs in the comparisons of CKvsM100.

In this study, a total of 551 TFs were annotated, belonging to 43 TFs families in rosemary suspension cells (Figure 7A). Under the MeJA treatments, many differentially expressed TFs were found (Figure 7B,C). For example, MYB (15.97%), AP2-EREBP (14.88%), bHLH (8.17%), NAC (7.62%), WRKY (5.63%), G2-like (4.54%), C2H2 (4.54%), MADS (3.45%), Tify (3.09%), C3H (2.90%) and LOB (2.90%) which frequency were more than 2.90%, indicating that these TFs might play important regulatory roles in the synthesis of rosemary suspension cells important metabolites and the stress-response.

In order to understand the differentially expressed TFs in rosemary suspension cells, there were 320, 305 and 434 differentially expressed TFs in the CKvsM10, CKvsM50 and CKvsM100 comparisons, respectively (Figure 7D–F). The classification of differentially expressed TFs by family showed that MYB (48, 15.00%), AP2-EREBP (38, 11.88%), WRKY (28, 8.75%), G2-like (20, 6.25%), bHLH (20, 6.25%) in the CKvsM10 comparison; MYB (48, 15.74%), AP2-EREBP (42, 13.77%), NAC (30, 9.84%), WRKY (21, 6.89%), bHLH (21, 6.89%) in the CKvsM50 comparison; MYB (61, 15.72%), AP2-EREBP (57, 14.69%), bHLH (38, 9.79%), NAC (35, 9.02%), WRKY (22, 5.67%) in the CKvsM100 comparison. There were more differentially expressed TFs in the CKvsM100 than the CKvsM10 and CKvsM50 comparisons. Indicating that, MYB was the most significantly differentially expressed TFs in rosemary suspension cells responding to MeJA, AP2-EREBP, bHLH and WRKY might play an important role in rosemary suspension cells responding to different concentrations of MeJA through differential expression or specific expression.

### 3.10. qRT-PCR Verification of DEGs Related to MeJA

**Figure 8:**
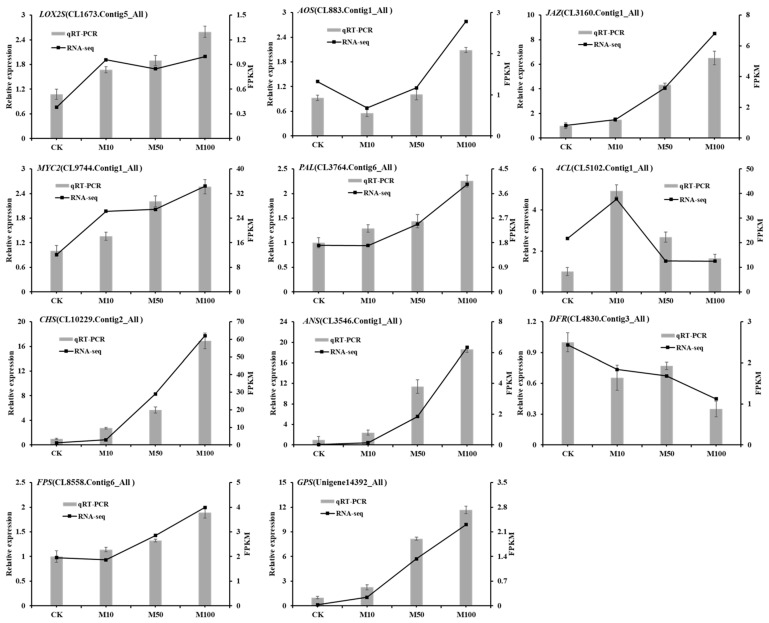
qRT-PCR verification of DEGs in rosemary suspension cells responding to MeJA.

In order to further verify the reliability of gene expression profiles obtained by RNA-seq, the expression pattern of 11 DEGs was estimated by qRT-PCR in rosemary suspension cells under different concentrations of MeJA (Figure 8). The expression of *LOX2S*, *AOS*, *JAZ*, *MYC2*, *PAL*, *CHS*, *ANS*, *FPS* and *GPS* were the highest in the 100 μM treatment, only the expression of *AOS* was the lower in the 10 μM than the 0 μM MeJA (CK) treatment, while the expression of *4CL* was the highest in the 10 μM treatment. The expression pattern of *DFR* was the highest in 0 μM MeJA, followed by 50, 10 and 100 μM MeJA treatments. In summary, the qPCR verification results showed that the relative expression of the genes was similar to the transcriptome, affirming the consistency of RNA-seq data and our results.

## 4. Discussion

### 4.1. Phenylpropanoid and Terpenoid Pathway Were Closely Related to the Synthesis of Important Metabolite in Rosemary Suspension Cells

In our study, phenylpropanoid and terpenoid backbone biosynthesis pathways were significantly enriched. Phenylpropanoid biosynthesis was closely related to the biosynthesis of flavonoids and rosmarinic acid. Exogenous MeJA regulated phytoalexin and polyphenol biosynthesis by up-regulating *PAL* in *Arabidopsis* [36]. PAL was the key rate limiting enzyme, its activity was enhanced after plant stress [37]. In plants, MVA and MEP pathway provided premises for terpenoids biosynthesis [38]. During the process of rosemary suspension cells under different concentrations of MeJA, a total of 157, 35 and 143 DEGs involved in the phenylpropane, flavonoids and terpenoids biosynthesis, respectively, mostly were up-regulated. The key synthase genes *FDPS* and *GGPS* of precursors as GPP were up-regulated in rosemary suspension cells under 100 μM MeJA, which was similar to the results of *Taxus* and *Chrysanthemum indicum* [39,40]. Therefore, MeJA might enhance phenylpropane and terpenoids biosynthesis by regulating the DEGs in the phenylpropanoid and terpenoids biosynthesis pathways. In summary, those DEGs might involve in regulating secondary metabolites, such as flavonoids, rosmarinic acid and carnosic acid.

### 4.2. ROS Scavenging Systems Improved Tolerance of Rosemary Suspension Cells Responding to MeJA

After the external environment changed, the balance was destroyed and ROS was overproduced to damage the plants, which could induce the synthesis of phytoalexin [41,42]. Plant cells mainly respond to stress through enzymatic and non-enzymatic antioxidant systems [43,44,45]. In *Arabidopsis*, 177 PLP enzymes might regulate the expression of genes of hormone synthesis and signal transduction, *AtPDX2* could be induced under light, drought and low-temperature stress [46,47,48]. In *Arabidopsis vtc1* mutant, the content of Asc was 70% lower than the wild type, while the content of ABA and the expression of *NCED* were significantly increased [49]. Asc played an important role in protecting the body and avoiding normal metabolism from oxidative stress [50,51,52]. After PEG-6000 and H_2_O_2_ treatments, the antioxidant enzyme system and secondary metabolites synergistically eliminated excess ROS in *S.baicalensis* [53,54]. Proline confers tolerance to Cd-stress in tobacco *BY-2* cells by different mechanisms [55]. Different type of stresses stimulated plants to secrete flavonoids [56,57]. In our study, the activities of SOD, CAT, POD, PPO and PAL were significantly increased, the contents of H_2_O_2_ and MDA were reduced in rosemary suspension cells under different concentrations of MeJA. These phenomena improved the repair capacity of oxidative damage and ROS scavenging ability in plant cells and thereby enhancing the ability to cope with external factors in plant [58]. The changes of enzymatic and non-enzymatic antioxidants by MeJA could prevent oxidative damage, and promote the accumulation of secondary metabolites, such as flavonoids in rosemary suspension cells. 

### 4.3. Plant Hormone Signal Transduction Played a Key Role in Rosemary Suspension Cells Responding to MeJA 

Plant hormones play an important role in the synthesis of secondary metabolites, and the regulatory process involves a variety of signal transduction and interaction factors [59]. In *Arabidopsis arf6* and *arf8* single mutants and sesquimutants, *ARF6* and *ARF8* gene dosage affected the accumulation of JA and the expression of *MYB* [60]. Over-expression *miR393* could stabilize *ARF1* and *ARF9* to increase glucosinolate and decrease camalexin in *Arabidopsis* [61]. Exogenous cytokinin and ethylene up-regulated the alkaloid production through independent pathways in periwinkle suspension cells [62]. Exogenous ABA, GA and ethylene increased the levels of phenolic acids by activating the PAL and TAT in *Salvia miltiorrhiza* hairy roots [63]. DELLA would inhibit the transcriptional activation of downstream target genes by binding to PIF3 and PIF4 [64,65,66]. In *Tripterygium wilfordii* suspension cells, the contents of triptolide and triptolide increased significantly with exogenous ABA after 10 days [67], and the expression of *AACT*, *MCT*, *CMK* and *CYP450* in terpenoids biosynthesis increased significantly by exogenous MeJA [68]. The content of total essential oil was significantly increased by exogenous BR in *Mentha canadensis* [69]. Under MeJA treatment, the expression of *ODC*, *ADC*, *PMT*, *QPRT* and *bHLH* in nicotine biosynthesis were up-regulated, which affected the accumulation of nicotine and other pyridine organisms in *Nicotiana tabacum* [70]. *JAZ8* participated in the biosynthesis of phenolic acids in *Salvia miltiorrhiza* under MeJA treatment [71]. Over-expression *ORCA3* enhanced the expression of *Tdc*, *Str*, and *D4h* in terpenoid biosynthetic genes increased the accumulation of terpenoid indole alkaloids in *Catharanthus roseus* [72,73]. MeJA induced the expression of JA biosynthesis and signaling pathway genes, and the transcription factor *MYC2* was released from JAZ to transcribe and activate the down-stream response genes in *Arabidopsis* [74,75,76], *Hevea brasiliensis* [77], *Nicotiana tabacum* [78], *Catharanthus roseus* [79], *Chrysanthemum indicum* [80] and *Vitis vinifera* [81]. In our study, many genes showed differential expressions under MeJA treatment, e.g., *ARF*, *DELLA*, *EIN3*, *JAZ*, *MYC2*, further study of these genes is required. Thus, 100 μM MeJA could significantly induce the genes of JA biosynthesis and signal transduction and inhibit certain hormone signal transduction, especially SA signal transduction. MeJA could regulate the biosynthesis of secondary metabolites via the complex network of plant hormone signal transduction in rosemary suspension cells, such as terpenoids, flavonoids.

### 4.4. Transcription Factors Played an Important Role in Rosemary Suspension Cells Responding to MeJA

TFs played a key role in plant growth, development, secondary metabolism and resistance to stress [82]. In *Salvia miltiorrhiza*, *PAP1* could induce the expression of *PAL*, *C4H* and *CHS* to promote the accumulation of anthocyanins [83], *MYB39* and *MYB4* could negatively regulate the synthesis of rosmarinic acid [84,85], *ERF115* had different regulatory effects on salvianolic acid and tanshinone in different tissues and organs [86]. *MYC2* mediated plant responding to stress, regulated the genes in the biosynthesis of many secondary metabolites [87]. AP2/ERF, bHLH, MYB, NAC, WRKY and bZIP TFs families were related to the metabolism of terpenoids, MYB and bHLH could induce plant anthocyanin accumulation [88,89]. In *Arabidopsis*, several R2R3-MYB were involved in the regulation of flavonoid biosynthesis [90,91,92], *MYB4* negative controlled sinapate ester biosynthesis through down-regulated *C4H* in a UV-dependent manner [93], *MYB11*, *-12*, *-111* regulated flavonol biosynthesis by up-regulated *CHS*, *CHI*, *F3H*, *F3’H* and *FLS* [90,94], *MYB75*, *-90*, *-113*, *-114* controlled anthocyanin biosynthesis in vegetative [95], *MYB123* controlled the biosynthesis of proanthocyanidins in the seed coat [96]. *MYB5*, *-14* played a key role in seed coat polymer biosynthesis in *Medicago truncatula* [97]. *OsWRKY13* was an important TF to resist the infection of rice blast fungus by directly or indirectly regulating the genes of SA and JA signaling pathway [98]. Over-expression *li049*, the expressions of genes related to lignan biosynthesis were significantly up-regulated and the content was increased in *Isatis indigotica* hairy root [99]. *TcDRREB* regulated the key gene of the paclitaxel synthesis pathway to increase the content of paclitaxel increased with JA treatment in *Taxus* suspension cells [100]. DELLA could interact with JAZ1, reduce the inhibition of *JAZ1* to *MYC2* in *Arabidopsis* [101]. *RIM1* was a negative regulator for JA signaling in *Rice* [102]. In our study, the classification of them showed that MYB was most related to MeJA. R2R3-MYB were differentially expressed under MeJA treatment, *MYB111* was significantly up-regulated under 50 and 100 μM MeJA, and down-regulated under 10 μM MeJA. Therefore, MeJA might play a regulatory role in the synthesis of rosmarinic acid, terpenoids, flavonoids and other metabolites by including the MYB, AP2-EREBP, *MYC2* and *DELLA* in rosemary suspension cells.

**Figure 9:**
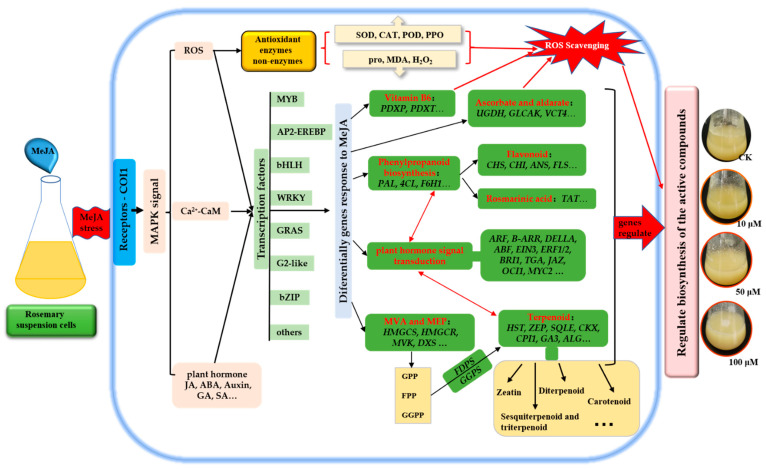
Schematic representation of rosemary suspension cells responding to MeJA. Antioxidant enzymes and non-enzymes, DEGs of many biosynthesis pathways played an essential beneficial in rosemary suspension cells responding to MeJA. Red indicates the key biosynthesis and signals transduction pathways, italics indicate key DEGs.

In conclusion, MeJA increased the activities of PAL, SOD, POD, CAT and PPO, and reduced the contents of H_2_O_2_ and MDA, thus accelerated the ROS scavenging. A comparative analysis of global gene expression patterns provided subsets of DEGs in rosemary suspension cells under MeJA treatment. Our study revealed the expression profiles of genes involved in plant hormones signaling pathway, flavonoids, terpenoid backbone and phenylpropanoid biosynthesis pathway, TFs, indicating their regulatory role in the synthesis of the active compounds in rosemary suspension cells under MeJA treatment. We suggested a feasible working model based on the result (Figure 9). These transcriptomic data provided new insights into future functional studies, as a means of studying the molecular mechanisms on the biosynthesis of active compounds and the mining of key enzyme genes in rosemary suspension cells.

## Data Availability

Not applicable.

## Supplementary Materials

The following Supplementary Materials are available online at https://www.mdpi.com/article/10.3390/genes13010067/s1, Figure S1: Species distribution of the Nr database, Figure S2: GO Functional classification, Figure S3: KOG Functional classification, Figure S4: KEGG assignment of Unigenes, Figure S5: Top 20 enriched and regulated KEGG pathways, Figure S6: Rosemary suspension cells primary and secondary metabolic pathways under different concentrations of MeJA, Figure S7: DEGs assigned to plant hormone signal transduction pathway under MeJA treatments, Figure S8: DEGs assigned to Phenylpropanoid biosynthesis pathway under MeJA treatment, Figure S9: DEGs assigned to Terpenoid biosynthesis pathway under MeJA treatment, Table S1: Primers used for real-time quantitative PCR, Table S2: Summary of the sequencing data in each sample, Table S3: All_Unigene length distribution statistics, Table S4: The statistical resscriptome assembly, Table S5: The statistical resscriptome assembly, Table S6. Gene expression values for by FPKM, Table S7. The top 20 most expressed genes (FPKM) form CK, M10, M50, M100 library, Table S8: Statistical analysis of differential gene annotations to GOs in different comparison Groups.
