# Supplementary material for: Transcriptome Analysis Reveals Differentially Expressed Genes That Regulate Biosynthesis of the Active Compounds with Methyl Jasmonate in Rosemary Suspension Cells
Source: Genes (Basel). 2021 Dec 27;13(1):67. doi: 10.3390/genes13010067 (PMC8775320; doi:10.3390/genes13010067)
Supplement: Supplementary file 1 [file genes-13-00067-s001.zip › genes-1466509-supplementary.pdf]

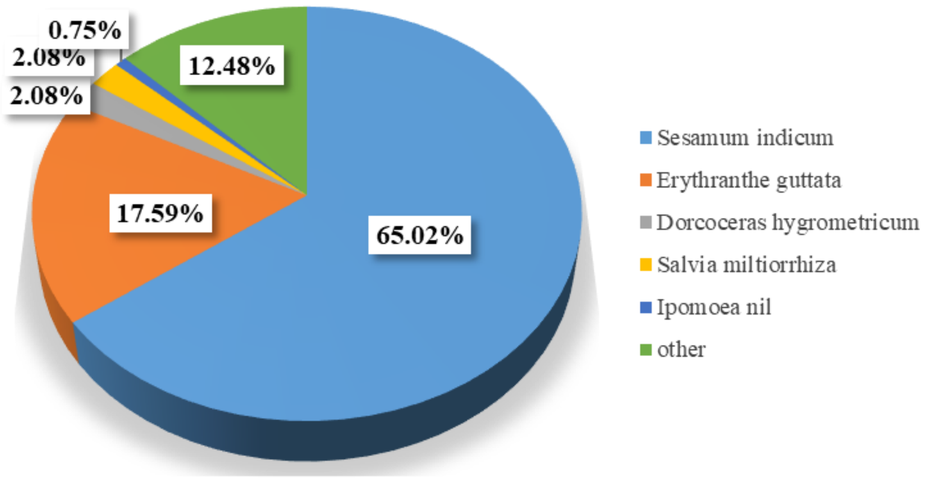

Figure S1. Species distribution of the Nr database

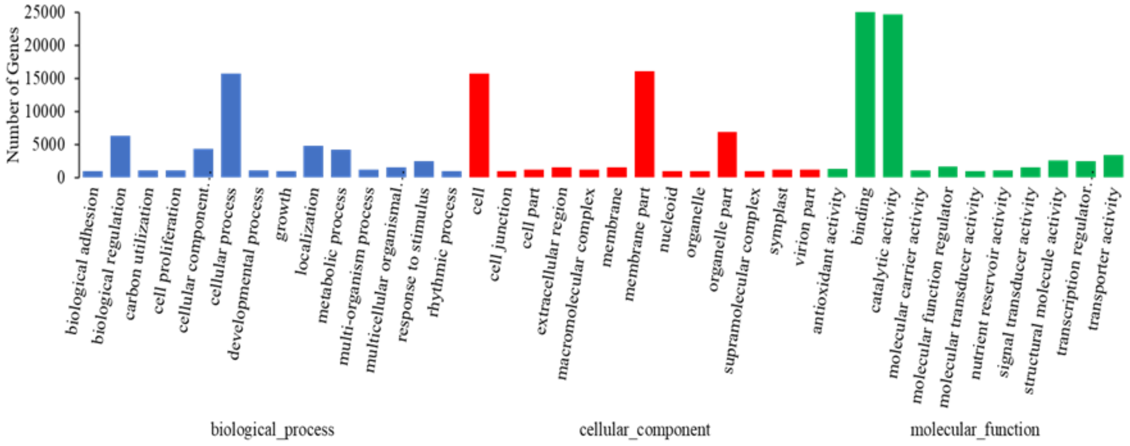

Figure S2. GO Functional classification

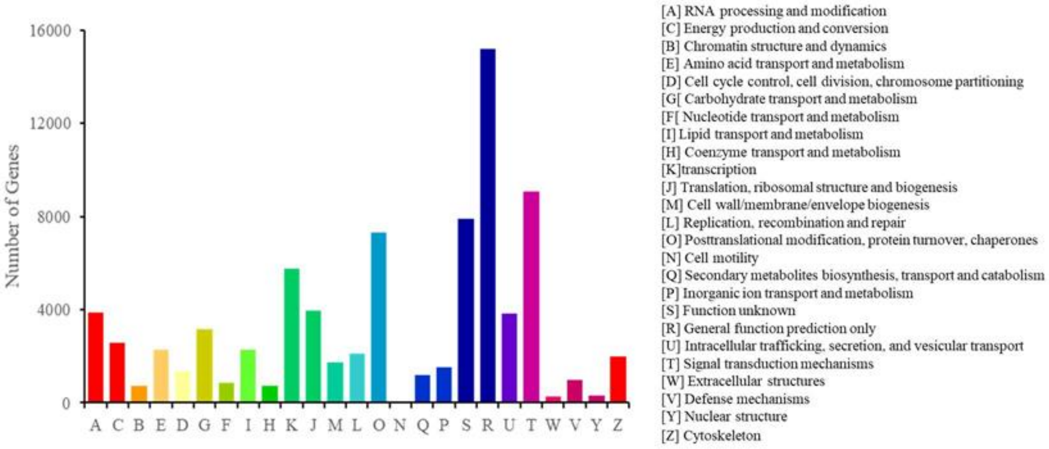

Figure S3. KOG Functional classification

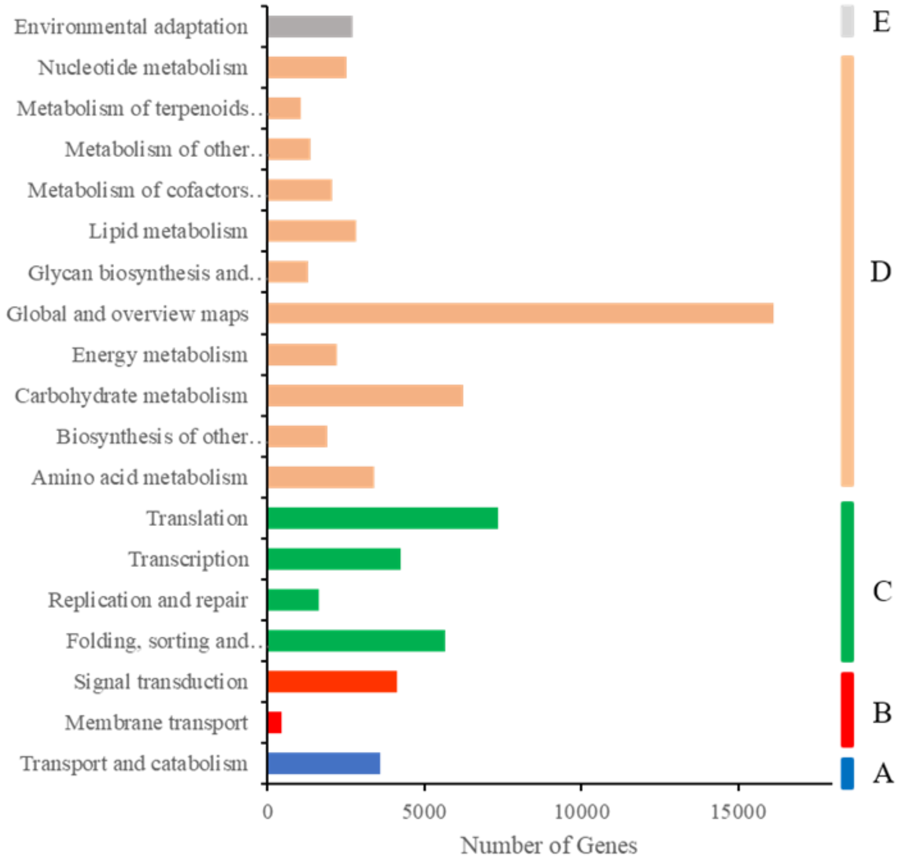

Figure S4. KEGG assignment of Unigenes. A, Cellular Processes. B, Environmental Information Processes. C, Genetic Information Processes. D, Metabolism E, Organismal Systems.

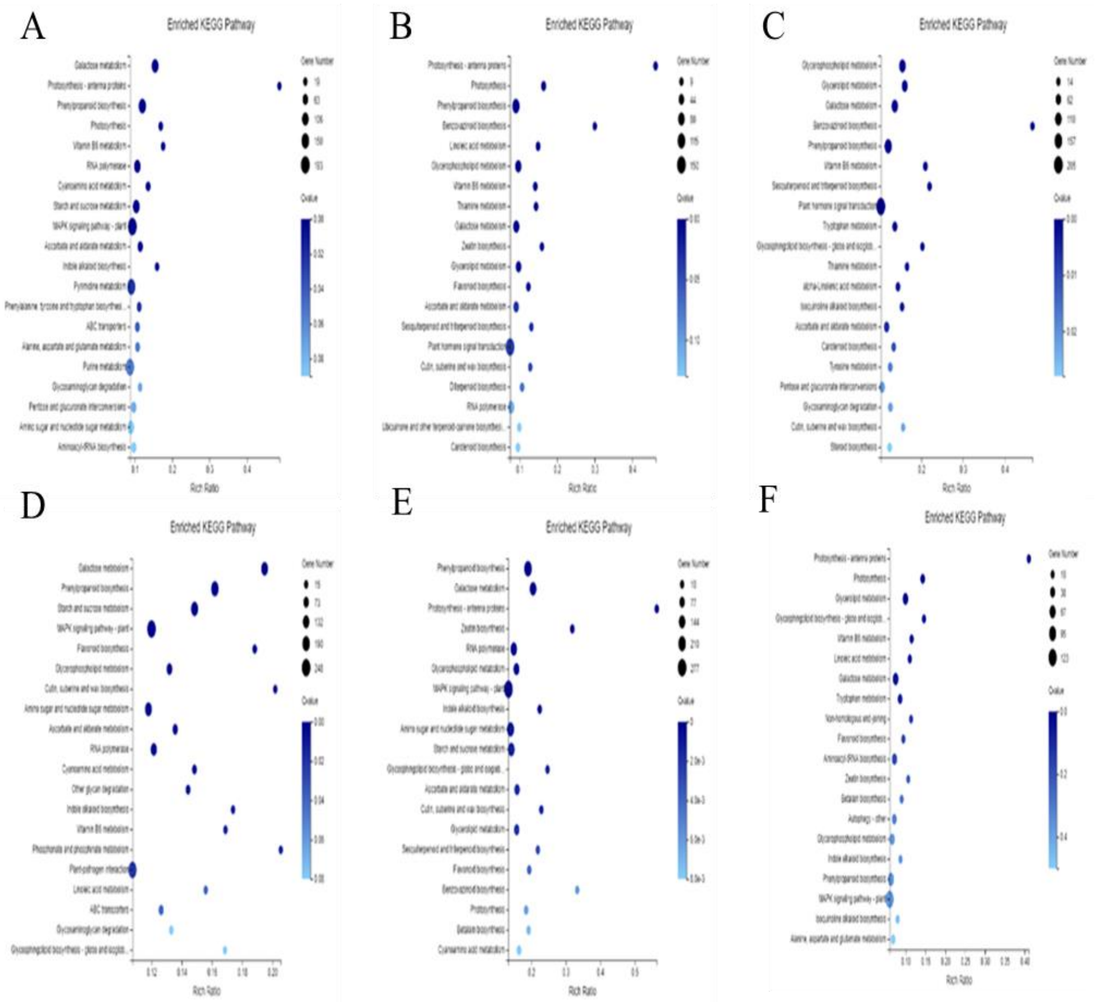

Figure S5. Top 20 enriched and regulated KEGG pathways. A, CKvsM10. B,CKvsM50. C,CKvsM100. D, M10vsM50.E, M10vsM100. F, M50vsM100. The Y-axis on the left represents the KEGG pathways, and the X-axis indicates the rich factor. low q-values are shown in dark blue, and high q-values are depicted in light blue.

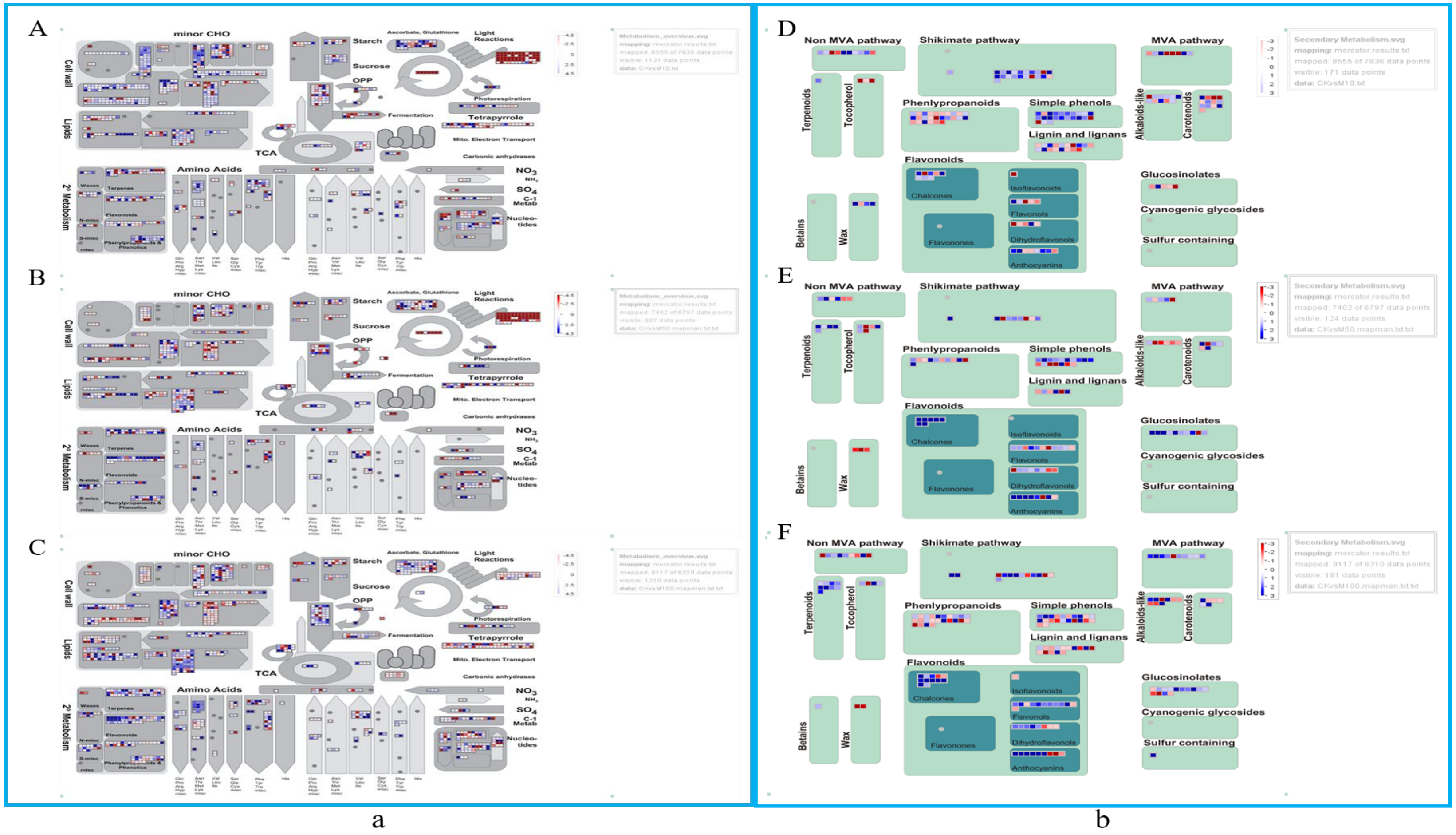

Figure S6. Rosemary suspension cells primary and secondary metabolic pathways under different concentrations of MeJA: (a) Primary metabolic pathways; (b) Secondary metabolic pathways. A, CKvsM10. B, CKvsM50. C, CKvsM100. D, CKvsM10. E, CKvsM50. F, CKvsM100.

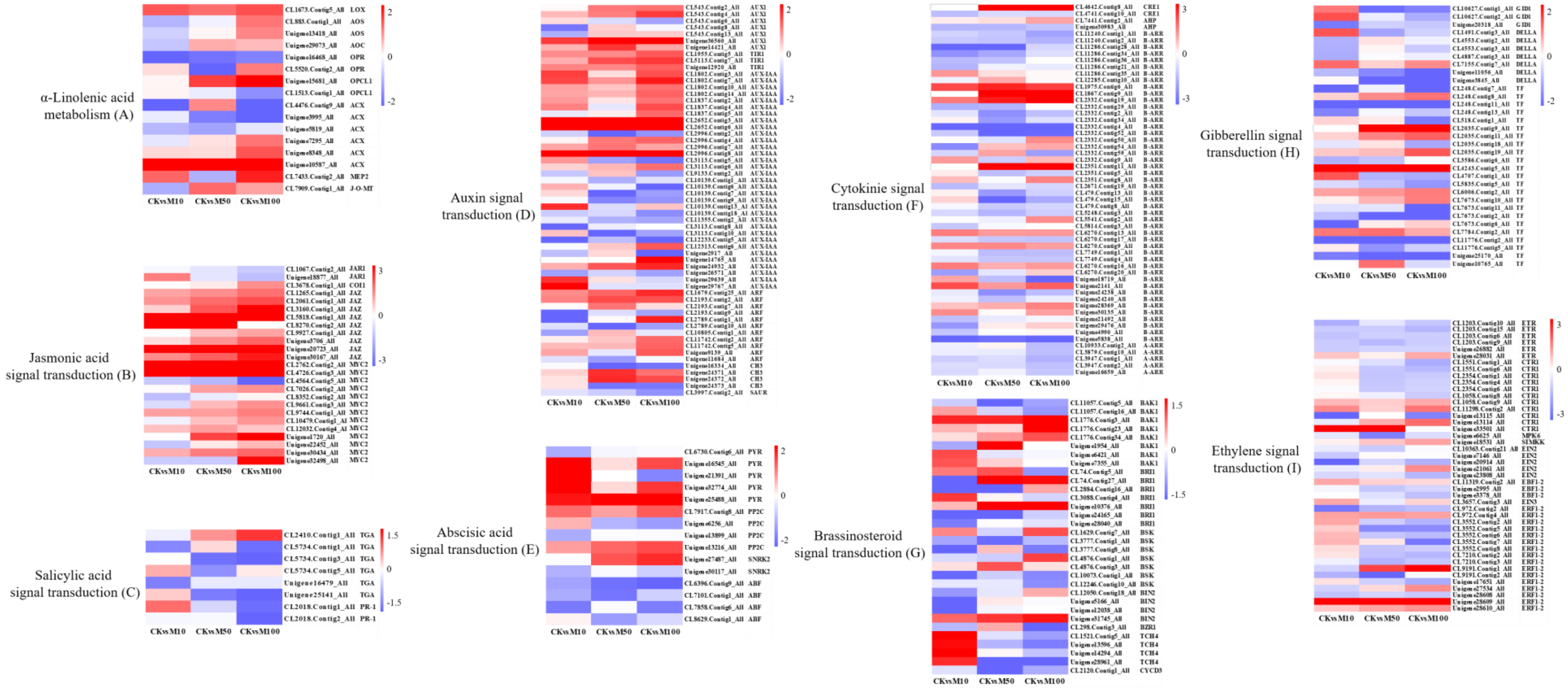

Figure S7. DEGs assigned to plant hormone signal transduction pathway under MeJA treatments. A, α-Linolenic acid metabolism (JA biosynthesis); B, Jasmonic acid signal transduction; C, Salicylic acid signal transduction; D, Auxin signal transduction; E, Abscisic acid signal transduction; F, Cytokinie signal transduction; G, Cytokinie signal transduction; H, Gibberellin signal transduction; I, Ethylene signal transduction. From the red to the blue corresponds to the numerical value of Log_2 Fold Change (CKVSM10), Log2 Fold Change (CKVSM50) or Log2 FoldChange (CKVSM100) from the high to the low.

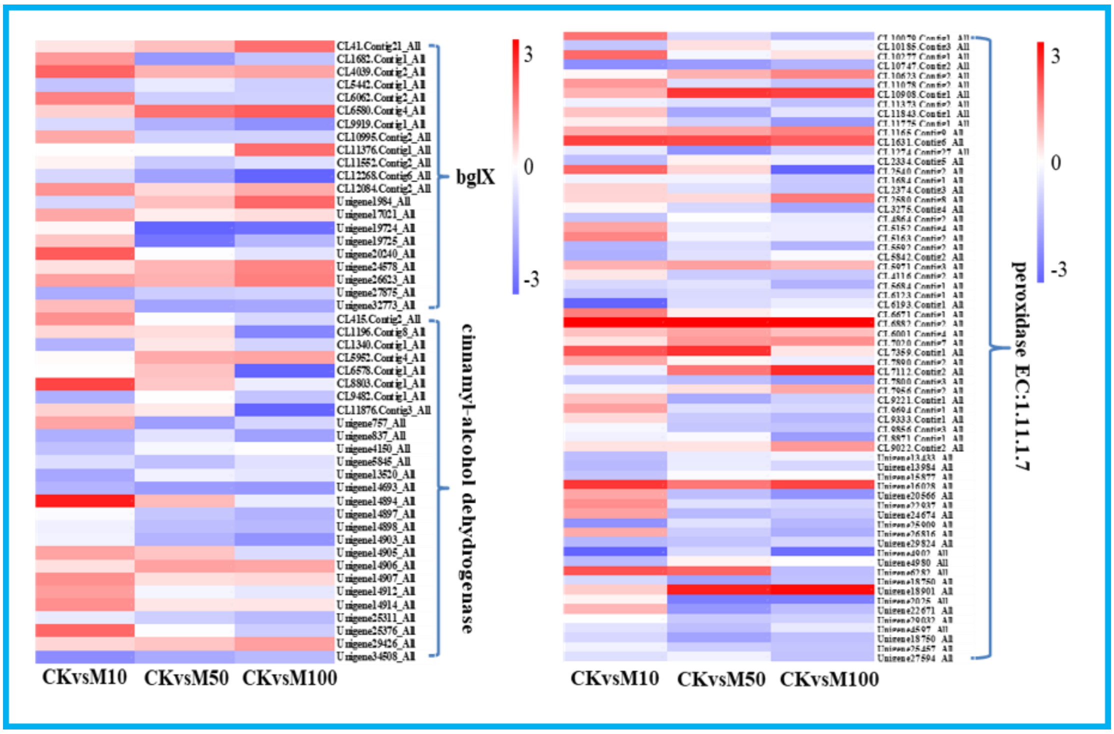

Figure S8. DEGs assigned to Phenylpropanoid biosynthesis pathway under MeJA treatment.

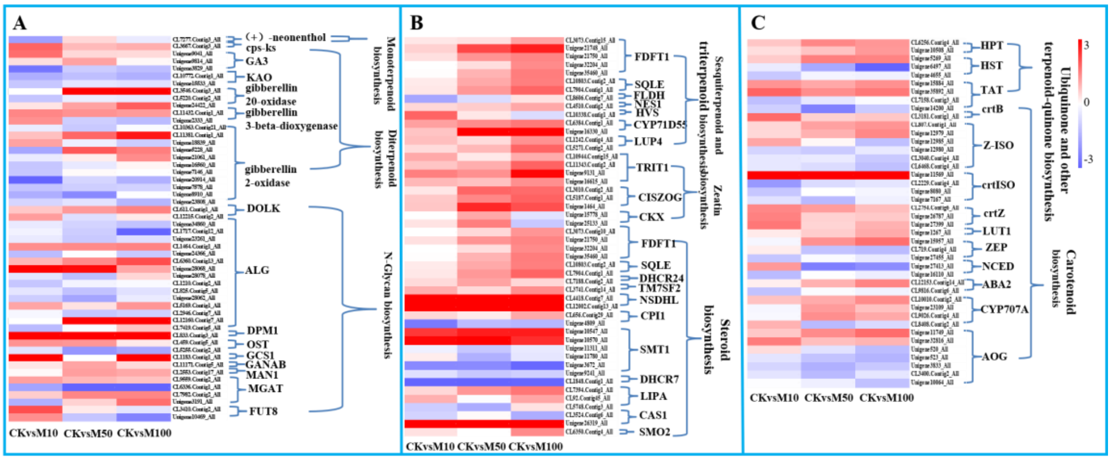

Figure S9. DEGs assigned to Terpenoid biosynthesis pathway under MeJA treatment. A: A heat map of the expression of DEGs related to Monoterpenoid biosynthesis, Diterpenoid biosynthesis, N-Glycan biosynthesis pathway; B: A heat map of the expression of DEGs related to Sesquiterpenoid and triterpenoid biosynthesis, Zeatin biosynthesis, Steroid biosynthesis pathway; C: A heat map of the expression of DEGs related to Ubiquinone and other terpenoid-quinone biosynthesis, Carotenoid biosynthesis pathway.

Table S1. Primers used for real-time quantitative PCR.

Gene_id	Perimer	Primer sequence (5' to 3')
CL1673.Contig5_All	*LOX2S*-q-F	AGACGCGCTTCTCATATGGG
	LOX2S -q-R	TTGTGTGTCGAGGTTAGGCC
CL883.Contig1_All	*AOS*-q-F	TGTAGACATCCGGTGCCAAC
	AOS-q-R	GATCTCTCCGCCGTAGCATTG
CL3160.Contig1_All	*JAZ*-q-F	CCTCTAACCGCAGACCGATC
	JAZ-q-R	TCATCAGGCCGGTACTAGCT
CL9744.Contig1_All	*MYC2*q-F	TCGCTCCCCGCAGAAATTAG
	MYC2-q-R	CGCTCTTCATCTCGGCAAGA
CL3764.Contig6_All	*PAL*-q-F	AGCAACAAGAGCAGCAATGC
	PAL-q-R	GCGATGTAGGAGAGAGGCACG
CL5102.Contig1_All	*4CL*-q-F	AGGGCCCGATGGTAACTTTG
	4CL-q-R	CATAGCGAGGACGTGATGCTG
CL10229.Contig2_All	*CHS*-q-F	CGATACATGCACCTGACGGA
	CHS-q-R	ACTCCTTGATGGCCTTCTGCG
CL3546.Contig1_All	*ANS*-q-F	GAATTCACGATCACGCGGTG
	ANS-q-R	TGAAAACGTCTCAGGCCTCCA
CL4830.Contig3_All	*DFR*-q-F	TCTTGCCTGCTGTCATGTGT
	DFR-q-R	GACAGCTGCAGAGAGAAGG
CL8558.Contig6_All	*FDPS*-q-F	TCTCTCATCCTCGGAAGGCA
	FDPS-q-R	GTGTGGAAGTGGAGGAGTGG
Unigene14392_All	*GGPS*-q-F	CTCCCATGCCAATCCCAACT
	GGPS-q-R	TGTTGAGGTAGGCGTCGATG

Table S2. Summary of the sequencing data in each sample.

Sample	Total Raw Reads (M)	Total Clean Reads	Total Clean Bases(Gb)	Clean Reads Q20(%)	Clean Reads Q30(%)	Clean Reads Ratio(%)
CK_1	72.69	69,880,812	6.99	97.62	89.86	96.13
CK_2	70.19	67,478,396	6.75	97.59	89.81	96.14
CK_3	72.69	69,399,868	6.94	97.66	89.98	95.46
M10_1	72.69	69,219,724	6.92	97.64	89.95	95.22
M10_2	72.69	69,315,798	6.93	97.57	89.66	95.36
M10_3	70.18	67,092,804	6.71	97.69	90.12	95.59
M50_1	72.69	69,391,412	6.94	97.69	90.11	95.46
M50_2	72.69	69,402,678	6.94	97.70	90.15	95.48
M50_3	72.69	69,101,312	6.91	97.60	89.86	95.06
M100_1	75.2	71,191,500	7.12	97.40	89.30	94.67
M100_2	72.69	69,364,484	6.94	97.66	90.03	95.42
M100_3	69.71	66,314,608	6.63	97.68	90.10	95.13

Table S3. All_Unigene length distribution statistics

Sequence length	200-500	500-1000	1000-1500	1500-2000	2000-2500	2500-3000	>=3000
Number	19345	24216	22373	18727	12558	7818	11033
Proportion	16.67	20.86	19.28	16.13	10.82	6.74	9.51

Table S4. The statistical resscriptome assembly

Sample	Total Number	Total Length	Mean Length	N50	N70	N90	GC(%)
CK_1	59690	67711539	1134	1669	1154	556	43.66
CK_2	59058	66494837	1125	1645	1139	551	43.69
CK_3	59260	67270226	1135	1668	1153	555	43.64
M10_1	57904	66262777	1144	1673	1165	563	43.66
M10_2	58816	67279586	1143	1673	1162	563	43.59
M10_3	54688	61945359	1132	1657	1153	556	43.87
M50_1	59996	66919306	1115	1623	1125	545	43.66
M50_2	59055	67083426	1135	1662	1151	554	43.66
M50_3	59133	66736466	1128	1644	1135	555	43.71
M100_1	64402	71579893	1111	1614	1120	546	43.49
M100_2	61824	70936638	1147	1673	1162	564	43.49
M100_3	59873	67423284	1126	1650	1137	548	43.62
All-Unigene	116067	177816828	1532	2069	1516	848	43.03

Table S5. The statistical resscriptome assembly

Values	Number	Percentage
Nr	99,842	86.02%
Nt	82,519	71.10%
Swissprot	75,796	65.30%
KEGG	80,961	69.75%
KOG	81,035	69.82%
Pfam	77,595	66.85%
GO	53,718	46.28%
Intersection	32,359	27.88%
Overall	101,986	87.87%
Total	116,067	100 %

Table S6. Gene expression values for by FPKM.

FPKM Interval	CK	M10	M50	M100
FPKM≤0.1	4281(3.99%)	4628(4.31%)	4442(4.14%)	4156(3.86%)
0.1<FPKM≤1	26671(24.81%)	27451(25.56%)	26899(25.05%)	29985(23.99%)
1< FPKM<10	62520(58.16%)	60803(56.62%)	62094(57.83%)	63721(59.19%)
10≤FPKM< 100	13283(12.35%)	13078(12.12%)	13103(12.20%)	13152(12.22%)
FPKM≥100	818(0.76%)	802(0.75%)	828(0.77%)	788(0.73%)

Table S7. The top 20 most expressed genes (FPKM) form CK, M10, M50, M100 library.

NO	Gene_id	FPKM_CK	Description
1	CL91.Contig2_All	3813.54	defensin-like cystein-rich peptide
2	CL5934.Contig9_All	2490.66	extracellular ribonuclease LE
3	CL2557.Contig19_All	2130.46	PREDICTED: titin-like
4	CL604.Contig2_All	1517.01	extensin-3
5	CL11203.Contig5_All	1422.12	major pollen allergen Lol p 11-like
6	CL11865.Contig24_All	1389.98	Hypothetical protein SELMODRAFT _431225
7	CL6276.Contig1_All	1258.25	hypothetical protein NitaMp027
8	CL7735.Contig5_All	1214.65	aquaporin-like protein, partial
9	CL9730.Contig2_All	1084.79	21 kDa protein-like
10	Unigene1082_All	965.99	unknow
11	CL3979.Contig4_All	925.06	pathogen-related protein STH-2
12	CL8010.Contig3_All	906.69	putative metallothionin 2a
13	CL3979.Contig3_All	863.58	pathogen-related protein STH-2
14	Unigene27255_All	853.25	unknow
15	CL10207.Contig1_All	803.39	metallothionein
16	CL1901.Contig2_All	769.66	hypothetical protein MNEG_14416
17	CL5971.Contig7_All	710.45	LOW QUALITY PROTEIN: protein E6-like
18	CL155.Contig3_All	708.39	glycine-rich RNA-binding protein GRP1A-like
19	CL10411.Contig1_All	695.24	2-alkenal reductase (NADP(+)-dependent)
20	CL301.Contig11_All	682.1	uncharacterized protein LOC110601548
**NO**	**Gene_id**	**FPKM_M10**	**Description**
1	CL91.Contig2_All	3293.52	defensin-like cystein-rich peptide
2	CL2557.Contig19_All	2648.08	PREDICTED: titin-like
3	CL11203.Contig5_All	2014.59	major pollen allergen Lol p 11-like
4	CL604.Contig2_All	1786.69	extensin-3
5	CL5934.Contig9_All	1709.20	extracellular ribonuclease LE
6	CL11865.Contig24_All	1441.91	hypothetical protein SELMODRAFT_431225
7	CL3979.Contig4_All	1420.08	pathogen-related protein STH-2
8	CL3979.Contig3_All	1232.53	pathogen-related protein STH-2

9	CL6276.Contig1_All	1229.28	hypothetical protein NitaMp027
10	CL7735.Contig5_All	1228.61	aquaporin-like protein, partial
11	Unigene1082_All	1103.99	unknow
12	CL3284.Contig1_All	981.343	major allergen Pru ar 1-like
13	CL1631.Contig24_All	913.69	cationic peroxidase 1
14	CL9730.Contig2_All	907.87	21 kDa protein-like
15	CL11203.Contig4_All	886.08	major pollen allergen Lol p 11-like
16	Unigene11436_All	858.08	xyloglucan endotransglucosylase /hydrolase protein 15-like
17	CL4003.Contig2_All	842.48	PREDICTED: snakin-2-like
18	Unigene22583_All	816.77	hypothetical protein MIMGU_mgv1a016790mg
19	CL1631.Contig25_All	809.593	cationic peroxidase 1
20	CL1901.Contig2_All	781.42	hypothetical protein MNEG_14416
**NO**	**Gene_id**	**FPKM_M50**	**Description**
1	CL91.Contig2_All	3843.50	defensin-like cystein-rich peptide
2	CL5934.Contig9_All	3293.01	extracellular ribonuclease LE
3	CL2557.Contig19_All	2880.15	PREDICTED: titin-like
4	CL11865.Contig24_All	1701.58	hypothetical protein SELMODRAFT_431225
5	CL7735.Contig5_All	1651.29	aquaporin-like protein, partial
6	CL11203.Contig5_All	1511.69	major pollen allergen Lol p 11-like
7	CL604.Contig2_All	1379.56	extensin-3
8	CL3979.Contig4_All	1097.86	pathogen-related protein STH-2
9	CL9730.Contig2_All	1084.93	21 kDa protein-like
10	CL6276.Contig1_All	1065.23	hypothetical protein NitaMp027
11	CL3979.Contig3_All	988.44	pathogen-related protein STH-2
12	CL8010.Contig3_All	937.07	putative metallothionin 2a
13	CL5971.Contig7_All	802.59	LOW QUALITY PROTEIN: protein E6-like
14	Unigene1082_All	798.46	unknow
15	CL10411.Contig1_All	785.49	2-alkenal reductase (NADP(+)-dependent)
16	CL2557.Contig3_All	739.48	PREDICTED: titin-like
17	CL1231.Contig1_All	727.55	catalase isozyme 3 isoform X2
18	CL301.Contig11_All	722.35	uncharacterized protein LOC110601548
19	CL3284.Contig1_All	715.98	major allergen Pru ar 1-like
20	CL5971.Contig4_All	662.68	LOW QUALITY PROTEIN: protein E6-like
**NO**	**Gene_id**	**FPKM_M100**	**Description**
1	CL5934.Contig9_All	4293.72	extracellular ribonuclease LE
2	CL91.Contig2_All	3059.08	defensin-like cystein-rich peptide
3	CL2557.Contig19_All	2888.16	PREDICTED: titin-like
4	CL11865.Contig24_All	1848.79	hypothetical protein SELMODRAFT_431225
5	CL604.Contig2_All	1733.49	extensin-3
6	CL7735.Contig5_All	1431.61	aquaporin-like protein, partial

7	CL11203.Contig5_All	1377.14	major pollen allergen Lol p 11-like
8	CL3979.Contig4_All	1199.19	pathogen-related protein STH-2
9	CL1231.Contig1_All	1135.85	catalase isozyme 3 isoform X2
10	CL8010.Contig3_All	1086.51	putative metallothionin 2a
11	CL9730.Contig2_All	1083.53	21 kDa protein-like
12	CL3979.Contig3_All	1073.88	pathogen-related protein STH-2
13	CL6276.Contig1_All	992.68	hypothetical protein NitaMp027
14	Unigene26825_All	960.71	acidic endochitinase SE2-like
15	CL2557.Contig3_All	852.80	PREDICTED: titin-like
16	Unigene34674_All	807.90	hypothetical protein CCACVL1_24177
17	CL4003.Contig2_All	787.86	PREDICTED: snakin-2-like
18	CL3284.Contig1_All	713.37	major allergen Pru ar 1-like
19	CL7343.Contig6_All	711.97	glycine-rich RNA-binding protein GRP1A-like
20	Unigene1082_All	708.63	unknow

Table S8. Statistical analysis of differential gene annotations to GOs in different comparison Groups.

group	Biological process	Cellular component	Molecular function	Up-Regulated	Down-Regulated	Total
CKvsM10	1912	2296	3029	1884	2053	3937
CKvsM50	1576	1814	2457	1505	1657	3162
CKvsM100	1938	2199	3120	2163	1805	3968
M10vsM50	2356	2828	3785	2426	2508	4934
M10vsM100	2794	3295	4444	3089	2710	5799
M50vsM100	1282	1483	2039	1451	1156	2607
